# Emotion Regulation in Schema Therapy and Dialectical Behavior Therapy

**DOI:** 10.3389/fpsyg.2016.01373

**Published:** 2016-09-14

**Authors:** Eva Fassbinder, Ulrich Schweiger, Desiree Martius, Odette Brand-de Wilde, Arnoud Arntz

**Affiliations:** ^1^Department of Psychiatry and Psychotherapy, University of LuebeckLuebeck, Germany; ^2^De Viersprong, Netherlands Institute of Personality DisordersHalsteren, Netherlands; ^3^Department of Clinical Psychology, University of AmsterdamAmsterdam, Netherlands

**Keywords:** emotion regulation, emotional avoidance, Schema therapy, dialectical behavior therapy, experiential techniques, skills

## Abstract

Schema therapy (ST) and dialectical behavior therapy (DBT) have both shown to be effective treatment methods especially for borderline personality disorder. Both, ST and DBT, have their roots in cognitive behavioral therapy and aim at helping patient to deal with emotional dysregulation. However, there are major differences in the terminology, explanatory models and techniques used in the both methods. This article gives an overview of the major therapeutic techniques used in ST and DBT with respect to emotion regulation and systematically puts them in the context of James Gross' process model of emotion regulation. Similarities and differences of the two methods are highlighted and illustrated with a case example. A core difference of the two approaches is that DBT directly focusses on the acquisition of emotion regulation skills, whereas ST does seldom address emotion regulation directly. All DBT-modules (mindfulness, distress tolerance, emotion regulation, interpersonal effectiveness) are intended to improve emotion regulation skills and patients are encouraged to train these skills on a regular basis. DBT assumes that improved skills and skills use will result in better emotion regulation. In ST problems in emotion regulation are seen as a consequence of adverse early experiences (e.g., lack of safe attachment, childhood abuse or emotional neglect). These negative experiences have led to unprocessed psychological traumas and fear of emotions and result in attempts to avoid emotions and dysfunctional meta-cognitive schemas about the meaning of emotions. ST assumes that when these underlying problems are addressed, emotion regulation improves. Major ST techniques for trauma processing, emotional avoidance and dysregulation are limited reparenting, empathic confrontation and experiential techniques like chair dialogs and imagery rescripting.

## Introduction

Dialectical behavior therapy (DBT) and Schema therapy (ST) have both shown to be effective treatment methods especially for borderline personality disorder (BPD) (Zanarini, 2009; Stoffers et al., 2012), a disorder that is specially associated with emotional dysregulation. Although both, ST and DBT, have a cognitive-behavioral background, there are major differences in how both methods deal with emotions and emotion dysregulation. This paper provides an overview of background and theory of both treatment approaches, a model how both methods conceptualize emotion dysregulation and the major therapeutic techniques with respect to emotion regulation. Further it is discussed how DBT and ST concepts and techniques map onto the process model of emotion regulation from James Gross (Gross, 2015). Similarities and differences of the two methods are highlighted and illustrated with a case example.

## Background and theory

### Dialectical behavior therapy (DBT)– background and theory

#### Development of dialectical behavior therapy and the dialectic of acceptance and change

DBT was developed in the late 1980s by Linehan (1993a,b), originally for chronically (para)suicidal patients, then extended to patients with BPD. To that time, these patients had been considered as “untreatable.” A focus on problem solving or cognitive restructuring, according to standard cognitive behavioral therapy (CBT), had been experienced as potentially invalidating by the patients and had led to frustration, angry reactions, resistance and treatment drop outs. On the other side, focusing on acceptance and validation has also been perceived as problematic by patients since their problems and behaviors did not change. This led to one of the most important features of DBT, the “*dialectic” of acceptance and change*. This means, that therapists, on the one hand accept patient as they are and provide validation for their thoughts, emotions and behaviors, while on the other hand therapists acknowledge the need for change and foster the learning of new skills to deal with problems and to reach personal goals (Linehan and Wilks, 2015). This dialectic stance has been inspired by principles of dialectic philosophy (e.g., everything is transient and finite, everything is composed of contradictions, passage of quantitative into qualitative changes, change results from a helical cycle of thesis, antithesis and synthesis).

DBT is currently the most extensively studied and used approach to treat BPD (Stoffers et al., 2012). In addition, DBT has been adapted and successfully tested for BPD with several comorbidities and other psychiatric conditions in which problems in emotion regulation lead to psychopathology such as substance misuse (Linehan et al., 1999, 2002; Dimeff and Linehan, 2008), eating disorder (Safer et al., 2001; Telch et al., 2001; Kröger et al., 2010), post-traumatic stress disorder (Steil et al., 2011; Harned et al., 2012, 2014; Bohus et al., 2013), or depression (Lynch et al., 2007).

#### Skill acquisition and the four modules in DBT

DBT conceives emotion regulation skills deficits as the core of BPD. Thus, the main focus of the treatment is the acquisition of a functional emotion regulation. With its CBT background, DBT draws from a broad spectrum of cognitive and behavioral treatment techniques to induce the development of skills in emotion regulation. Skill training is embedded in four modules:

- *Mindfulness* is central to all skills in DBT. The mindfulness skills derive from traditional Buddhist meditation practice, though they do not involve any religious concepts. In DBT it means the practice of being fully aware and present in the present moment, experiencing one's emotions, thoughts or body sensations without judging and without reacting to them. The mindfulness skills are divided into “what skills” (observing, describing and participating) and “how-skills” (non-judgmentally, one-mindfully and effectively). An important concept of this module is “wise mind,” which allows to base decision making on a balance between intuition and facts. The implicit goal is to provide the experience that emotions and cognitions are internal events that are a patterned response to external and internal stimuli. Mindfulness allows watching cognitions and emotions from an observer perspective as separate both from the external world and the self.
- *Emotion regulation* compromises detailed psychoeducation on emotions in general and a broad spectrum of specific emotions to foster an in depth understanding of emotions and emotion regulation. It teaches skills in problem solving, checking reality and taking opposite action to behavioral tendencies associated with specific emotions as well as skills reducing emotional vulnerability. The module intends to give the patient a fresh look on emotions and to decrease emotional and experiential avoidance. A critical feature is to enable the patient to make an active choice between acting with an emotion or opposite to it.
- *Interpersonal effectiveness* teaches how to obtain objectives skillfully and how to act effectively with respect to objectives, relationship and self-respect. The implicit objective is to reduce interpersonal avoidance which is the key to change experiential and emotional avoidance and to increase interpersonal behavior that has a high probability of being positively reinforced.
- *Distress tolerance* focusses on teaching crisis survival skills. It fosters acceptance in situations that cannot be otherwise changed or avoided without making things worse. There is an emphasis on self-soothing, improving the moment and adaptive distraction. Important concepts are “radical acceptance” and “willingness.” The module intends to decrease self-destructive ways of emotional avoidance like self-injury, substance abuse or distraction with risk taking behavior.

#### Major components of DBT

In standard DBT there are four *major components*: skills training group, individual psychotherapy, telephone coaching, and consultation team.

- *DBT skills training group* is usually carried out in a group format with approximately eight patients and two skills trainers. The group follows a manualized protocol (Linehan, 2015a,b). In the original format group members meet once a week for approximately two and a half hours, yet there are varying adaptations to heterogeneous settings. The skills training group focusses on psychoeducation and training of behavioral skills in the four DBT modules mindfulness, interpersonal effectiveness, emotion regulation and distress tolerance. Homework assignments for patients are given every session and aim at practicing the learnt skills in everyday live.
- *DBT individual psychotherapy* is carried out by an individual therapist on a weekly basis with 50 min sessions. The individual therapist is the primary treatment provider and responsible for treatment planning, crisis management and decisions about individual modifications of treatment. The individual therapist supports the patient in the implementation of the skills, he has acquired in the skills training group, helps with trouble shooting and removing obstacles to change and ensures generalization of change. The individual therapy follows a hierarchy with four stages and structured target levels for each stage. The idea is to optimize the change process and to begin the change process with reducing life-threatening and therapy interfering behavior and then proceed to support skills acquisition, treatment of comorbid conditions, finding solutions for problems in living and creating a life worth living. Basic treatment strategies comprise specific dialectical strategies, validation, behavior analysis, didactic strategies and problem solving, commitment strategies, contingency management, observing-limits procedures, skills training, exposure-based procedures, cognitive modification and stylistic strategies like reciprocal communication and case management strategies.
- *DBT telephone coaching:* In crisis situation patients can call their individual therapist outside the sessions and receive support in applying suitable skills. It was designed to help generalize skills into the patient's daily life.
- *DBT therapist consultation team:* Communication between the providers of individual therapy and skills training is very important to support each other in providing the treatment. In standard DBT the therapists meet weekly and review which skills are currently the focus of the group sessions and discuss any problems the patients have in applying the skills. The meetings safeguard that the therapists share a common language and a common knowledge about the skills communicated to the patients. Further they support each other to provide DBT.

### Schema therapy (ST)–background and theory

#### Development of ST

Schema therapy also derives from CBT and was originally developed by Young et al. (2003) for patients, which did not respond to standard CBT. These patients often had a comorbid personality disorder (PD) and showed complex, rigid, and chronic psychological problems in emotion regulation and in interpersonal relationships, which in most cases could be followed back into their childhood. These problems also impaired the psychotherapeutic process as those patients had difficulties in forming a collaborative relationship with the therapist and could not be reached with standard CBT techniques due to (anticipated) intensive emotional reactions and coping strategies such as avoidance or surrender. In the process of finding ways to address the needs of these patients, Young integrated ideas and techniques from other theoretical orientations into a classical CBT frame (especially attachment theory, Gestalt therapy). A strong emphasis was placed on the biographical aspects for the development of maladaptive psychological patterns through traumatization in childhood and frustration of basic childhood needs. The therapeutic relationship was conceptualized as “*limited reparenting”* meaning that the therapist creates an active, caring, parent-like relationship with the patient (Young et al., 2003).

ST was developed as a transdiagnostic approach, but also provides disorder specific models for most PDs (see overview in Arntz and Jacob, 2012). Several studies have shown that treatment based on that model is very effective for patients with BPD (Giesen-Bloo et al., 2006; Farrell et al., 2009; Nadort et al., 2009; Dickhaut and Arntz, 2013), but also for other PDs (Bamelis et al., 2013). Good results are also reported for depression, post-traumatic stress disorder, eating disorders, and complex obsessive compulsive disorders (Cockram et al., 2010; Simpson et al., 2010; Malogiannis et al., 2014; Renner et al., 2016; Thiel et al., 2016).

#### Central concepts in ST: schemas, coping strategies and modes

ST is based on the idea that aversive experiences and frustration of basic childhood needs (e.g., safety, love, attention, acceptance, or autonomy) lead in interaction with biological and cultural factors to the development of *maladaptive schemas.* Schemas are defined as organized patterns of information processing compromising thoughts, emotions, memories, and attention preferences (Young et al., 2003). Schemas have a strong impact on how individuals view themselves, their relationships to others and the world. Young described 18 maladaptive schemas, e.g., shame/defectiveness, social isolation, mistrust, or unrelenting standards (Young et al., 2003). If a maladaptive schema gets activated, associated painful emotions arise. In order to deal with these intensive emotions, *coping strategies* (surrender, avoidance, overcompensation) are developed that attenuate aversive emotions but impair adaptive interpersonal and self-regulatory behavior.

While working with BPD patients Young discovered that the schema model was not optimal to explain and work with the quick mood and behavior changes of these patients. Thus, he extended the schema theory with the mode model approach, first for BPD later for narcissistic patients (Young et al., 2003). Since then, the mode model has be elaborated and empirically tested with specific mode models for most PDs (Lobbestael et al., 2008, 2010; Bamelis et al., 2011). A mode is a combination of activated schemas and coping strategies and describes the current emotional-cognitive-behavioral state. A mode can change quickly, while a schema is rigid and enduring (schema = trait, mode = state; Young et al., 2003). It is therefore a convenient concept in clinical practice as it helps patients and therapists understand the sometimes quick emotional changes.

Modes can be divided into 4 broad categories:

- *Dysfunctional child modes* are activated when patients experience intense aversive emotions, e.g., fear or abandonment, helplessness, sadness (vulnerable child modes), anger, or impulsivity (angry/impulsive child modes). Child modes develop when major needs, particularly attachment needs, were frustrated in childhood.
- *Dysfunctional parent modes (punitive or demanding)* are associated with self-devaluation, feelings of self-hatred, guilt, shame, or extremely high standards. They reflect internalized negative beliefs about the self, which the patient has acquired in childhood due to the behavior and reactions of significant others (e.g., parents, teachers, peers).
- *Dysfunctional coping modes* describe the excessive use of the coping strategies surrender (Compliant surrender mode), avoidance (e.g., Detached protector mode or Self-soother mode), or overcompensation (e.g., Self-Aggrandizer mode or Bully-and-Attack-mode) in order to reduce the emotional pain of child and parent modes. These modes are usually acquired early in childhood to protect the child from further harm and are therefore considered as “survival strategies.”
- The *healthy modes* of the *healthy adult mode* and the *happy child mode* represent functional states. In the healthy adult mode, people can deal with emotions, solve problems and create healthy relationships. They are aware of their needs, possibilities and limitations and act in accordance with their values, needs and goals. The happy child mode is associated with joy, fun, play, and spontaneity. The healthy modes are usually weak at the beginning of therapy.

For a detailed description of all modes see Arntz and Jacob (2012).

#### Therapy goals and treatment strategies in ST

The major goal in ST is helping patients to understand their emotional core needs and learn ways of getting needs met in an adaptive manner or to help them deal with the frustration if needs cannot be satisfied. This requires breaking through long-standing emotional, cognitive and behavioral patterns, meaning change of dysfunctional schemas, coping strategies and modes. According to the mode model there are specific goals connected with every mode guiding the treatment: Child modes are supported and comforted. Dysfunctional parent modes are reduced, therapists even “combat” the punitive parent mode. Dysfunctional coping modes should be reduced and replaced by healthier, more flexible strategies. However, as these modes have served as “protective shield” for vulnerable child modes for such a long time, therapists have to proceed particularly careful. Only if the patient feels safe enough in the therapeutic relationship, the adaptive function of the coping modes has been validated enough and their advantages as well as disadvantages have been reviewed cautiously, the patient will be able to reduce his “protective shield” and learn healthier strategies to deal with emotions and relationships. A last important goal is to strengthen the healthy modes. To achieve these goals, *mode-specific cognitive, experiential, and behavioral interventions* are used, with a strong emphasis on experiential techniques like chair dialogs and imagery rescripting. “*Limited reparenting”* (behaving like a “good parent” toward the patient, within the boundaries of the therapy relationship) is central to ST and underlies all therapeutic techniques. “Limited reparenting” serves as an antidote to traumatic experiences and leads to corrective emotional experiences. “Limited reparenting” provides empathy, warmth, protection and care for the patient. However, it may also be necessary to set limits to the patient and to empathically confront him with the consequences of his behavior and the need to change.

## DBT and ST models of emotion and emotion dysregulation

### How DBT conceptualizes emotion dysregulation

DBT explains BPD and its symptoms as the consequence of a severe disorder in the emotion regulation system. The genesis of these emotion regulation skills deficits is explained by the interplay of biological factors, learning history and social context (biopsychosocial model). Symptoms such as self-injury, binge eating, alcohol abuse, dissociation, or impulsive behaviors are regarded as coping strategies for intense emotions. Thus, a primary goal of DBT is to teach patients skill to tolerate and regulate intensive emotions.

DBT provides intensive psychoeducation on emotions and the (evolutionary) adaptive value of emotions. Emotions are considered as complex, brief, involuntary, patterned, full-system responses to internal and external stimuli (Ekman and Davidson, 1994). The DBT model of emotion and emotion regulation contains six interacting subsystems (Linehan, 2015b):

- Emotional vulnerability factors
- Internal and external events that serve as emotional cues (e.g., prompting events)
- Appraisal and interpretations of cues
- Emotional response tendencies (including physiological, cognitive, experiential responses and action urges)
- Non-verbal and verbal expressive responses and actions
- After-effects of the initial emotion, including secondary emotions and after-effects of problem behavior like social isolation or problematic peer relationships.

All DBT-modules (mindfulness, distress tolerance, emotion regulation, interpersonal effectiveness) are intended to improve understanding of own and other's emotions and learning emotion regulation skills. Patients are encouraged to train these skills on a regular basis. DBT assumes that expert knowledge on emotions, improved skills and skills use will result in better emotion regulation.

Although, this model was originally developed for patients with BPD, DBT has been applied in many other psychiatric conditions with data suggesting effectiveness (see above). Thus, it can be seen and used as a transdiagnostic model for optimization of emotion regulation skills in other clinical populations as well as in healthy individuals.

### How ST conceptualizes emotion dysregulation

In ST problems in emotion regulation are mainly seen as a consequence of adverse early experiences (e.g., lack of safe attachment, childhood abuse or emotional neglect). Negative experiences in childhood have led to fear of emotions and result in attempts to avoid emotions and (intimate) relationships. Dysfunctional schemas about the self and relationships to other as well as about the meaning of emotions prevail (e.g., “Emotions always hurt” or “to show emotions is a weakness”). ST assumes that when these underlying problems are addressed, emotion regulation improves. Thus, emotion regulation is not directly addressed as in DBT and there is no explicit model of emotion and emotion dysregulation as in DBT. However, emotion dysregulation can be explained by the mode model.

In ST emotion regulation skills improve by experiencing safe attachment and validation of needs and emotions through the specific features of the therapeutic relationship (esp. “limited reparenting” and “empathic confrontation”) and being safely guided through emotional processes with experiential techniques (e.g., processing of traumatic experience with imagery rescripting). Further, experiential avoidance mainly displayed by the coping modes is challenged as it blocks access and fulfillment of the patient's needs leading to enduring aversive emotions. ST assumes that by using these strategies the patient's fear of emotions reduces, while willingness to overcome experiential avoidance increases.

### Gross' process model of emotion regulation and its connection to DBT and ST

Gross' modal model of emotion regulation is the currently prevailing generic model to describe the emotion generating process (Gross, 2015). It compromises a *situation—attention—appraisal—response—sequence*: Briefly, the emotion sequence begins with a psychologically relevant *situation*, which can emerge from the external environment (e.g., being criticized by the boss) or from internal triggers like thoughts, body sensations, or other emotions (e.g., having a thought like “I am a loser”). Such a situation draws the individual's *attention* (e.g., attention focus on angry eyes of the boss) and gives rise to an *appraisal* (e.g., “I am going to get fired”). It follows a response including biological/experiential (e.g., heart beating faster, flushing, feeling ashamed or anxious) and behavioral changes (e.g., looking down, apologizing for mistake). This response often changes the situation (e.g., boss feels sorry after apology and says “Well, besides that one mistake, I am very glad that we have you in the team”) and gives rise to a new sequence.

According to the “process model of emotion regulation” (Gross, 2015) emotions can be influenced by targeting any component of the situation—attention—appraisal—response—sequence:

- *Situation Selection* by avoiding or approaching situations (e.g., going to a party with nice people to make it more likely to have a feeling of joy or avoiding a critical person to avoid shame)
- *Situation Modification* refers to staying in the situation but adding new behavioral elements (e.g., by asking my friend to give me a hug)
- *Attentional Deployment* refers to selecting a new attentional focus within a situation (e.g., by talking to someone the feeling of shame gets stronger, if one focusses on own performance and mistakes like stuttering instead of shifting the attention focus to the conversation partner)
- *Cognitive Change* refers to modifying the appraisal of the situation or getting a critical distance to cognitions (e.g., saying: “The thought ‘I am a loser’ is a thought not the truth”)
- *Response Modulation* refers to directly influencing experiential, behavioral or physiological components of the emotional response [e.g., taking a deep breath to relax and calm down body sensations (modulation of biological response) or going to an exam although one is afraid to fail (modulation of action response)].

Table 1 gives an overview of the five categories of emotion regulation strategies from the Gross' process model and how DBT and ST concepts and techniques map onto the process model. This results in a theoretical framework that allows the discussion of similarities and differences of these two psychotherapeutic methods with respect to emotion regulation. It is important to note that in this table the primary association between related DBT or ST technique and category of emotion regulation strategies is mentioned, although many techniques address several categories of emotion regulation strategies. In the following we describe the concepts and techniques first for DBT and then for ST and make the connection to every category of the process model of emotion regulation.

**Table 1:** **Categories of emotion regulation strategies from the Gross' process model, related DBT, and ST concepts and techniques**.

**Categories of Gross' Process Model**	**DBT concept of emotion regulation difficulties**	**Related DBT techniques and skills**	**ST concept of emotion regulation difficulties**	**Related ST techniques**
Situation Selection	Interpersonal skills deficit Experiential Avoidance Deficit of reinforcement	Planned Activities: Accumulate Positive Emotions, Action to Build Mastery Interpersonal effectiveness	Schema avoidance Schema surrender Schema overcompensation Experiential Avoidance	Psychoeducation in terms of mode model to foster understanding and overcome schema coping/experiential avoidance Empathic confrontation of dysfunctional situation selection that repeats history (e.g., dysfunctional partner choice) Cognitive techniques (e.g., schema or mode diary's identifying triggers, situations and unhealthy coping). Behavioral techniques (e.g., role plays of present situations, planning of healthy steps, stopping unhealthy choices)
Situation Modification	Problem solving skills deficits Interpersonal skills deficit	Problem solving Interpersonal effectiveness skills	Dysfunctional modes vs. Healthy adult mode	Becoming aware of emotional needs and helping patient “modify” situation so that needs are better met. Behavioral techniques (e.g., role plays of present situations, problem solving) Imagery Rescripting and PsychoDrama (Modification of context and situation)
Attentional Deployment	Mindfulness skills deficits	Mindfulness	Schema or mode maintenance (as attention is focused on information that confirms schema/mode)	Attention shift to different modes (e.g., with chair dialog or cognitive techniques), esp. to healthy adult mode
Appraisal	Dialectical dilemmas, Experiential avoidance due to meta-belief on emotions	Dialectic thinking, Validation, Check the facts Reality Acceptance Managing dialectic dilemmas, Walking the middle path	Distortion of information by early maladaptive schemas, Dysfunctional modes vs. healthy adult mode	Identification and re-appraisal of schemas through cognitive techniques (e.g., schema or mode diaries, socratic dialoq, schema-dialog) and experiential techniques (e.g., chair work and imagery rescripting; including trauma reprocessing) Change of meaning of early experiences that underlie dysfunctional schemas
Response Modulation	High emotional reactivity and sensitivity, Emotion regulation skills deficits, Interpersonal skills deficits, Mindfulness skills deficit	**Biological/experiential response Modulation:** Change physiology (TIP skills) Self-soothing Half-Smile/Willing hand	Dysfunctional modes vs. healthy adult mode	Limited Reparenting (modeling and shaping of emotional response in direct contact) Helping to express emotions and needs Modeling of healthy ways to deal with emotions by therapist Empathic confrontation to block problematic emotional reactions and promote functional reactions Emotional exposure Imagery rescripting (processing of traumatic experiences, altering of emotional response) Behavioral techniques including alternative behavioral responses and healthy self-soothing, relaxing strategies.
		**Expression/action response modulation:** Opposite action Interpersonal effectiveness Exposure Behavioral techniques		
		**Managing emotional-after effects:** Psychoeducation on emotions Mindfulness and Acceptance skills (Identify and Label emotions, Observe and Describe Emotions, Exposure)		
		**Managing emotional vulnerability factors:** Change Biological Sensitivity (PLEASE-Skills)		

## Strategies and techniques to regulate emotions

### Concepts and techniques for emotion regulation in DBT

DBT is a treatment model developed for a clinical population. The process model of emotion regulation is a generic model developed in basic sciences two decades later (Neacsiu et al., 2015). Yet there is apparently a strong similarity between the conceptualizations of emotion regulation in the process model and DBT. DBT offers specific behavioral and cognitive strategies for the regulation of emotions in each category defined by the process model (Table 1). The DBT part of Table 1 was strongly inspired by Neacsiu et al. (2015), who already mapped the DBT model onto the process model of emotion regulation. DBT skills are taken from the recent DBT manual (Linehan, 2015a,b). As can be seen in the table the category of response modulation has been divided into *biological/experiential response* and *expression/action response*. Furthermore, we added *managing emotional after-effects of the initial emotion*, especially addressing secondary emotions, and *managing emotional vulnerability factors* to response modulation. In the following the main skills for each category are described. Of particular note is that especially mindfulness skills are essential for every category, since skills of each category afford mindful observing, describing and participating in the present moment effectively and without judgment.

#### Situation selection

Psychopathology related to this area arises when patient avoid situations that are important for their goals and values or if patients approach situations where they are more likely to have unpleasant emotions. Situational avoidance may result in a deficit of reinforcement and pleasant emotions. From the DBT perspective the difficulties in this area mainly arise from skills deficits resulting in experiential avoidance (e.g., the patient does not go to a date with a friend caring for her to avoid feelings of shame or anxiety). The alternative possibility is that patients with deficits in social cognitions or interpersonal effectiveness skills deficit do not avoid aversive situations that may be avoided without a penalty (e.g., the patient meets with an invalidating friend).

The skill “*Accumulating Positive Emotions*” teaches patients that by approaching pleasant situations or situations that are meaningful in the light of their values and goals, they can increase positive emotions and reinforcement in their daily life in the short (e.g., by creating more pleasant events) and in the long run by living a life fitting their own values. “*Building Mastery”* aims at engaging in activities that foster the sense of competence, self-control, and self-efficacy. These two skills have an important overlap with behavioral activation treatment for depression (Kanter et al., 2009) and are in line with the strong emphasis on values in acceptance and commitment therapy (ACT) (Hayes et al., 2012). Moreover, *interpersonal effectiveness skills* are trained with the patients, where they learn to anticipate the consequences of interpersonal situations (e.g., “if I go dancing, when I feel lonely and have drunken alcohol and flirt with a drunken, older man, there is a high probability that he will try to have sex with me”) and learn to take functional decisions, which situations to approach and which to avoid. Moreover, they learn how to build and maintain functional relationships and end destructing ones.

#### Situation modification

Successful modification of situations especially affords problem solving strategies and interpersonal effectiveness skills. Patients are taught steps of standard *problem solving* (D'Zurilla and Nezu, 1999) to find and execute effective and doable solutions and to reduce distress in problematic situations. Within *interpersonal effectiveness* patients learn how to reach their goal in a specific situation without hurting others or damaging their own self-respect.

#### Attentional deployment

Psychopathology in this area arises if the control of attention is inflexible and not directed to the situational context. Some patients avoid the perception of the situational context by distraction or dissociation because of fears of interpersonal rejection. Others focus their attention inwards on physical symptoms of anxiety or on internal cognitive processes like worry or rumination or try to suppress unwanted thoughts or emotions. *Mindfulness exercises* in DBT help to keep the focus of attention in the presence and to avoid inflexible attachment to internal events like thoughts and emotions.

#### Appraisal

Problems in this category are consequences of dysfunctional information processing. DBT assumes that patients have insufficient skills in dialectic thinking, that they do not consider sufficiently the opposing forces that make up inner and outer realities. Patients fluctuate between invalidation of their internal experience on the one side and states of cognitive fusion, when they treat interpretations, assumptions and thoughts and emotions as facts in the outer world on the other side (e.g., “If I am angry, he must have done something wrong”).

One core technique to teach dialectic thinking is *validation*. Using validation strategies, the therapist communicates to the patient that her behavior makes sense and has a connection to her present context and past learning history. This applies quite particularly when the behavior on the surface appears “dysfunctional.” Uncovering the validity within problem behavior is a crucial prerequisite for changing exactly this behavior. The repeated use of validation strategies by the therapist will finally result in patients using validation as a skill. The skill “*Check the facts”* is the dialectic counterpart. It is intended to help patients to view thoughts as thoughts and emotions as emotions and to disentangle thoughts and emotions from actual facts. With this skill patients learn to consider actual facts for their decisions. DBT uses *mindfulness skills* with their focus on the present context as an antidote to worry, rumination and threat monitoring.

DBT conceptualizes problems in the appraisal category as caused by dialectical dilemmas: Patients often show patterns of dichotomous thinking are stuck in polarities, unable to move to a synthesis and are unable to anticipate or accept change. The skill “*Walking the Middle Path*” from the module interpersonal effectiveness teaches patients to find a synthesis between opposites: e.g., to base decisions on facts while concurrently experiencing intense emotions, to have a strong desire for change while concurrently dealing in an accepting way with the present moment. Further psychopathology in this category arises from meta-beliefs patients have on emotions (e.g., “Emotions are bad and destructive” or “Emotions should always be trusted”). These meta-beliefs, in DBT called “*Myths about emotions,”* are challenged and psychoeducation is provided.

#### Response modulation

DBT explains problems in this area with high emotional reactivity and sensitivity as well as skills deficits in emotion regulation, interpersonal effectiveness, distress tolerance and mindfulness. In DBT it is very important to separate the *biological/experiential response* including the action tendency, the urge to act with the emotion, from the behavior response itself. One major DBT-skill “*opposite action*” aims acting opposite of the emotion-driven behavior (e.g., to approach a dog although you have dog fear and the emotion of fear tells you to avoid dogs). This skill is indicated when the emotion driven behavior is not in accordance with the facts or the values of the patient. Moreover, *managing emotional after effects* and *vulnerability factors* are important subcategories in DBT.

- **Biological/experiential response**
  For BPD patients emotions often come with a high intensity of aversive physical sensations causing a high distress and a strong action urge, DBT provides a set of *distress tolerance skills*. These skills aim to calm down the high physiological arousal and to block acting on maladaptive urges. For example, the “*TIP skills*” (abbreviation for: *T*ip your face into ice cold water, *I*ntense Exercise, *P*aced Breathing, and *P*aired Muscle Relaxation) teaches patients how to down-regulate their physiological response through temperature change, exercise, breathing, or muscle relaxation. For the down-regulation of distress there are many other strategies in the DBT manual including *self-soothing with the five senses, distracting* (e.g., with activities) or changing the body posture to a more accepting posture (e.g., *half smiling and willing hands*). Half smiling was developed in line with research showing that facial expression influence emotions (Ekman, 1993). Patients are supported to develop a “*distress tolerance skill chain”* for high distress situations and perform a sequence of distress tolerance skills (e.g., 1. Ice cold water, 2. run steps three times up and down 3. Bite into chili pepper). It is very important to acknowledge the dialectic that DBT aims at mindfully accepting arousal and emotions without judgment while at the same time acting to reduce arousal. These skills serve as crisis strategies in high distress situations to block dysfunctional action urges like self-injury, rage attacks, or alcohol consumption, which lead to a further complication of the situation.
- **Expression/action response**
  Every emotion comes with an action urge. Many times acting on the urge is effective. If the behavior does not fit the facts or the values of the patient, there is the possibility to modify the behavioral response. An important option for emotion regulation is “*opposite action*” where one explicitly does the opposite of the emotion-driven behavior. This skill of course includes *exposure* to unpleasant emotions and follows similar principles as exposure-based treatments for anxiety disorders (e.g., approaching the feared stimulus). These principles are transferred to other emotions such as shame, disgust, anger, guilt, or sadness. “Opposite action” is also an important part in the treatment of depression, as proposed by Behavioral Activation (Kanter et al., 2009): Patients are motivated to engage in activities and to act opposite to the depression urge of social withdrawal, inactivity and avoidance. Changing action tendencies includes changing the overt action (e.g., being kind to a person one is angry with) but also body language, facial expression, or tone of voice. To address all these components patients are also trained in *interpersonal effectiveness*. It is important to distinguish opposite action from thought or emotion suppression or submissive interpersonal behavior: Opposite action does not intend to suppress an emotion, but to be mindfully aware and accepting of an emotion and its action urge, but to be able to decide to act differently. Opposite action does not intend to “give in” in contentious issues, it opens up new possibilities for solving interpersonal conflict.
- **Emotional after-effects**
  Emotions do not only influence concurrent behavior, cognition and emotion but also future behaviors, cognitions and emotions. Therefore, emotional after-effects of events are an important topic for emotion regulation. After effects may give rise to specific changes in attention, physiology, behavior, and appraisal. Humans avoid or perform specific behaviors because they anticipate specific emotional states. Much of emotional distress is caused by secondary emotions due to judgments about the primary emotion (e.g., evaluation of anxiety as “stupid” leads to intensive shame as secondary emotion. The evaluation of anger as meaning “you are an aggressive person” leads to guilt when the primary emotion of anger occurs). Mindful awareness of these emotion cycles helps to interrupt them and to apply change strategies if necessary. In the first step DBT provides *psychoeducation on emotions* in general and on specific emotions such as anger, disgust, guilt, joy, love, shame, fear, envy, jealousy, or sadness. For each emotion the adaptive value, typical prompting events (cues), interpretations/appraisals, biological/experiential changes, expression/behavior changes, after-effects, and secondary emotions are explained and words how to describe the emotion are provided. This helps patients to “*identify and label emotions.*” Moreover, patients learn to *observe and describe* emotions in a non-judgmental way. A very important aspect is *mindfulness and acceptance* in *exposure to emotional experience*, especially to the primary emotions. This means “experiencing emotions without judging them or trying to inhibit them, distract from them or to hold on them” (Linehan, 2015b).
- **Emotional vulnerability**
  DBT aims at fostering resilience by addressing emotional vulnerability factors. PLEASE is an acronym for treating *P*hysical I*l*lness, balanced *E*ating, avoiding mood-*A*ltering substances, balancing *S*leep, and getting, adequate *E*xercise.

### Concepts and techniques for emotion regulation in ST

Although, emotion-oriented interventions and systematic emotional work are central to ST, it is important to keep in mind that in ST regulation of emotions is not in the foreground of ST theory. ST intends to change dysfunctional schemas on the self, on relationships to others and on the world as a whole (as well as on the meaning of emotions), which underlie today's problems. These dysfunctional schemas were developed early in childhood through adverse experiences and gave rise to coping strategies such as avoidance, surrender or overcompensation to deal with (expected) threat or gratification. These coping strategies have often become very rigid and block access to the underlying schemas. Thus, the schemas cannot be changed and the disorder is maintained. ST of course aims to break through these rigid coping mechanisms to reach the dysfunctional schemas, however the ultimate aim is to change the underlying schemas.

Painful emotions and difficulties in regulating them are seen as a consequence of these underlying dysfunctional schemas. For instance, if one's need for emotional intimacy cannot be met because the representation of other people includes that other people will take advantage of you, if they see this “weak” need, or will punish you for having this need, it is understandable that dysfunctional emotion regulation results (e.g., by keeping out of intimate relationships). If corrective experiences in treatment lead to a change of the schema representation of other people, then the problem is resolved. If the representation of emotion entails that emotions constitute a threat, the patient will feel unsafe with emotion, and use avoidance or overcompensation to prevent that emotions are triggered. The aim of ST then is to help the patient feel safer with emotions. Thus, the way patients view, experience and regulate emotions changes substantially through the course of treatment without emotion regulation being directly addressed.

To give a better overview on how emotions are worked with in ST we decided to present ST techniques with special regard to emotions first in line with the basic ST literature (Young et al., 2003; Arntz and Jacob, 2012) by dividing them in therapy relationship, experiential, cognitive, and behavioral techniques. Afterwards we explain how these concepts and techniques can be mapped to the process model of emotion regulation (see also Table 1).

#### ST techniques to work with emotions

##### Therapy relationship techniques

The therapy relationship is an important vehicle for corrective emotional and interpersonal experiences. The major techniques are “*limited reparenting”* and “*empathetic confrontation.”* With the central attitude of “*limited reparenting*” the therapist provides a good-parent-like relationship characterized by warmth, empathy, support, careful self-disclosure, and safe attachment. He helps the patient to become aware of his emotions and needs, gives support in expressing emotions and needs, validates them and—within certain boundaries—fulfills the needs. Especially important is the fulfillment of needs that were frustrated in childhood. “Limited reparenting” is specifically designed to serve as an antidote to the patient's maladaptive schemas. The therapist directly models and shapes the emotional response of the patient providing external emotion regulation like parents do for their children (e.g., if a patient feels anxious in a vulnerable child mode the therapist provides safety). Moreover, the therapist models healthy ways of intrinsic emotion regulation by showing how he deals with his own emotions and needs. “Limited reparenting” also means to help patients to experience emotions in a safe way without being overwhelmed by emotional distress. Thus, the therapist sets up emotional work in small steps and actively guides through the process.

With “*empathetic confrontation”* the therapist challenges experiential avoidance mainly displayed by the coping modes. He emphasizes the adaptive value of the coping mode, and at the same time makes clear that the coping mode blocks access and fulfillment of the patient's needs leading to enduring aversive emotions. Also he promotes functional emotional reactions. ST assumes that by using these strategies the patient's fear of emotions reduces, while willingness to overcome the coping modes increases and by this the pathway to heal dysfunctional schemas opens. In a way, ST-therapy relationship-strategies resemble the way how emotion regulation develops in children. In childhood extrinsic emotion regulation by caregivers is initially dominant (Gross, 2013; e.g., a sad child is soothed by its mother, who plays with the teddy bear for the child). By experiencing adaptive extrinsic emotion regulation by caregivers and getting models for intrinsic and extrinsic emotion regulation, children can learn intrinsic emotion regulation (e.g., the sad child soothes itself by playing with its teddy bear) and also extrinsic emotion regulation for others (e.g., the child soothes another sad child in kindergarten by playing with the teddy bear).

##### Cognitive techniques

Cognitive techniques compromise a range of techniques similar to the techniques also used in CBT. In regard to emotion regulation strategies patients receive intensive *psychoeducation on schemas, schema coping, modes, needs, emotions as well as on normal development of children*. Within the mode model the therapist illustrates, why and how coping modes developed and validates their function, which is mainly to shelter the child modes from more emotional pain. He explains what children need to develop a healthy way to deal with emotions and points out the differences to the patient's history (e.g., “when a child is angry, it is not okay to tell him, that it is egoistic and to withdraw affection. Every child would feel guilty then. The parent needs to talk to the child, to find out why it is angry and help the child to calm down.”). The therapist fosters *mode awareness*, in which emotions play an important role (e.g., “if I feel guilty I need to look if this feeling is connected to my punitive parent mode”). He explains the mode-specific goals of ST (e.g., fighting the punitive parent and soothing the child modes) and promotes mode change, best in the healthy adult mode. He helps with the *identification and re-appraisal of schemas and mode-related cognitive distortions* (e.g., identify “I am worthless” as a cognition of the punitive parent mode, restructuring from healthy adult mode). Other important cognitive techniques compromise *reviewing pros and cons* (e.g., of coping modes to overcome experiential avoidance) or *focusing long-term consequences* (e.g., “If I stay in the detached protector, it is not possible to get close to others and I will go on feeling lonely and depressive.”) or *writing diaries or flashcards* to promote mode awareness and mode change.

##### Experiential techniques

Experiential techniques including emotional processing of aversive childhood memories are extensively used and are central to ST, which is a main difference to standard CBT. The main focus of ST is on changing dysfunctional schemas and the meaning of emotions and needs through emotional restructuring. As such ST does not place a strong emphasis on typical CBT exposure techniques aiming at habituation and extinction. An emotion is processed until the respective emotion (i.e., sadness, loneliness) and the connected need (e.g., need for attachment) and if necessary its biographical background becomes clear, than the emotion can be restructured. The main experiential techniques are so-called “*chair dialogues*,” *imagery exercises*, most often *imagery rescripting*, and *historical role play.*

In *chair dialogs* different chairs are used for different perspectives or emotions. In ST, most often different modes are placed on different chairs and dialogs between them are performed. The patient changes the seats and expresses on every chair the perspective and emotions of the related mode. When another mode pops up, the therapist usually asks the patient to change the seat to the chair that symbolizes the popped-up mode (e.g., ‘I hear you have a strong feeling of loneliness. I think this is connected to your vulnerable child mode. Would you please take a seat on the vulnerable-child-chair and tell me how little Tanja feels?’). The therapist helps the patient to express his feelings and needs and to detect and experience different mode perspectives. The therapist might also model to express those perspectives, emotions and needs the patient finds hard to express. These exercises clarify ambivalent emotions and inner conflicts, which is an important diagnostic step to the solution of an emotional problem. Moreover, chair dialogs can be used to restructure modes and emotions leading to new emotional experiences and changes in the dysfunctional schemas, meaning of needs, and emotions. To achieve this, the therapist or the healthy adult mode addresses every mode by adapting his tone of voice, the content of what he says to the mode and his actions following the mode-specific goals of ST (e.g., comfort the vulnerable child mode, fighting the punitive parent mode). Thus, the patient experiences in a highly emotional way, that his needs and emotions are important and that self-devaluation can be reduced.

*Imagery exercises* can also be used for diagnostic reasons to clarify the biographical origin of dysfunctional schemas and emotional problems as well as related behavior patterns (*diagnostic imagery)*. Most often diagnostic imagery exercises start from a current situation associated with strong emotions. The patient is asked to image that situation with eyes closed, the therapist focusses especially on the emotions and where in the body the patient can feel the emotion. When the emotion is clear enough, the therapist asks the patient to wipe away the image of the current situation and just stay with the emotion (affect bridge) and go back to his childhood and see if an image that is associated to that emotion pops up. The childhood image is then again explored with emphasis on emotions and needs. *Imagery Rescripting* (Arntz and Weertman, 1999) is considered to be the most powerful technique to change schemas and the meaning of adverse childhood events and emotions. The patient is asked to image a stressful (childhood) memory related to his maladaptive schemas (e.g., emotional abuse). Such a situation can be found through affective bridges as explained above or can be directly taken from the reports of the patient. When the patient clearly feels the related emotions and needs, the “rescripting part” is started by introducing a helping figure in the image, which modifies the situation to a more pleasant ending for the child, meaning that the child's needs are fulfilled. This helping figure can be the patient himself in his healthy adult mode, if he is already strong enough. For patients with PD this is often not the case in the beginning of therapy. Thus, the therapist or another helpful person (even a fantasy figure) can be introduced as helping figure. In the “rescripting part” the needs of the child are fulfilled, meaning that the perpetrator is stopped and the child is protected and cared for. Aversive emotions such as anxiety, shame or guilt are reduced, while experiencing safety, secure attachment, warmth, love, joy, and other pleasant emotions are promoted. By this, the original meaning of the trauma is changed. For some patients rescripting works better in the form of a role play, for instance if imagery constitutes a problem. Note that from an ST-perspective it is not necessary that the patient relieves the whole trauma, since habituation is not the primary goal.

*Historical role-play* (Arntz and Weertman, 1999) is a form of drama therapy, where therapist and patient play a traumatic biographical memory together as a role play. The patient switches roles by playing his own role (most often as a child) in the first round and the role of the perpetrator (most often a parent) in the second. This helps the patient to see another perspective on the events and to change the meaning of the situation. If a patient e.g., feels unlovable, since his father did not show any interest and was annoyed by the child, the patient can see by overtaking the perspective of the parent, that the father was overwhelmed with work and had never learnt how to show feelings. By this he can understand that it is not him being unlovable, but the circumstances of the situation that made his father act like that.

##### Behavioral techniques

Behavioral techniques mainly aim at breaking through rigid behavior patterns connected with the coping modes. After many years of dysfunctional coping this behavior has often become habitual and patients lack other skills to deal with emotions and needs. Thus, they need support to learn new strategies. ST compromise a range of techniques similar to the techniques also used in CBT such as *behavioral experiments, role play, homework, planning of activities, problem solving*, or *skill training*. If pathological choices (e.g., of abusive partners, of abusive work situations) remain the therapist will also address this on a behavioral level (help patients make healthy choices what to avoid and what to approach). Often it is very hard or even impossible for patients to change their behavior in the beginning of therapy due to maladaptive schemas, thus these strategies have a stronger emphasis later in the course of therapy, and are often prepared by experiential techniques.

#### Connection of ST techniques to the james gross' process model

In the following we map the ST concepts and techniques on the James Gross' process model of emotion regulation by going through each category of emotion regulation strategies (see also Table 1).

##### Situation selection

Schema therapy explains why patients avoid situations that might be useful for them and do not leave situations that are harmful using the concepts of schema avoidance, schema surrender, and schema overcompensation. It is assumed that dysfunctional child, parent and coping modes are responsible for problematic avoidance behavior or inaction. ST uses psychoeducation about the mode model to help to understand and overcome problematic schematic coping and experiential avoidance. Empathetic confrontation is used to confront patients with dysfunctional *situation selection* that repeats history and by this maintains schemas (e.g., dysfunctional partner choice). Behavioral techniques like role plays of the present situation and actively changing what situations to select may be used to foster transfer of behavior from the therapy session into the life of the patient.

##### Situation modification

Similarly, ST assumes that problem solving skills that are necessary to improve situations may be blocked by schema avoidance, schema surrender or schema overcompensation. ST supports the patient to develop awareness of their modes and individual needs and helps patients to modify situations so that needs are better fulfilled. Behavioral techniques help with testing and transfer of problem solving skills. Cognitive techniques help to identify problematic situations, situational triggers and alternative ways to get needs met. Imagery rescripting and historical role play may in particular modify the internal context in problematic situations.

##### Attentional deployment

Dysfunctional schemas and modes are maintained, since attention is focused on information that confirms the dysfunctional schema or mode. This problem of attention that is inflexible and not directed to the situational context is addressed by ST using the attention shift that is associated with mode work through cognitive and experiential techniques. Chair dialogs for example require the patient to shift their attention to varying aspects of internal and interpersonal situations and facilitate the experience of the emotional changes associated with shifting attentional deployment.

##### Appraisal

One core assumption of ST is that information processing and decision making is influenced by early maladaptive schemas and that psychopathology is related to a dominance of dysfunctional modes to the detriment of the healthy adult mode. Consequently, when dealing with emotion regulation, appraisal is a core area for ST. Identification and re-appraisal of schemas through cognitive and experiential techniques are central for ST. ST assumes that mode awareness and cognitive flexibility that is developed during therapy allows the patient to switch from dysfunctional modes to the healthy adult mode and by this eliminate problematic appraisal processes. All experiential techniques promote *change of appraisal* especially through changing the meaning of emotions and early experiences that underlie schema.

##### Response modulation

ST assumes that psychopathology in this category is related to dysfunctional modes in particular dysfunctional child and parent modes and coping modes. The therapy relationship techniques, especially limited reparenting, aim to model and shape emotional responses in direct contact with the patient. Empathic confrontation is used to block problematic emotional reactions and promote healthy emotional reactions. Emotional exposure in experiential techniques is set up in small steps with shelter by the therapist and plays an important role of response modulation. In imagery rescripting traumatic experiences are processed and through the new script where the patient's needs get fulfilled the emotional response is directly altered. Behavioral techniques support the transfer of new responses into the everyday life of the patient.

### Similarities and differences between DBT and ST

Both treatments share a CBT background and help patients to deal with emotional dysregulation. Both explain development of emotional dysregulation with invalidating aversive experiences in childhood in interplay with biological factors even if later in the therapy process the biographical aspects play a more distinct role in ST. In both methods the therapeutic relationship is marked by validation, acceptance and warmth for patients and both treatments address experiential avoidance. However, there are major differences in the terminology, explanatory models and techniques used in both methods. Table 2 summarizes the main features, similarities and differences.

**Table 2:** **Main features, similarities, and differences of DBT and ST**.

	**DBT**	**ST**
Explanatory model	Emotion dysregulation as central problem, Biosocial theory to explain emotion dysregulation, Focus on connection between emotion regulation and dysfunctional behaviors	Case conceptualization using the mode concept; frustration of basic needs in childhood leads to the development of maladaptive schemas and modes, problems in emotion regulation and interpersonal relationships follow. Emotion dysregulation is not seen as the central problem
Integration of childhood experiences	No explicit focus except for psychoeducation and validation of emotional dysregulation	Full integration: Maladaptive schemas, today's problematic behaviors, fear of emotions and relationships are associated with biographical experiences; psychoeducation regarding basic needs of children
Trained skills	Primary aim is skill acquisition in the area of emotion regulation. Skills are trained in the four DBT-modules emotion regulation, distress tolerance, mindfulness and interpersonal effectiveness	Skills for emotion regulation are not directly trained. Fostering meta-understanding of the current mode, skills for using the healthy adult mode, awareness of one's own needs and ways to meet them
General therapeutic strategies	Validation strategies, explicit techniques in DBT (V1–V6) Dialectical strategies (balance between acceptance and change, pro-contra lists) Commitment strategies Skills training Extensive use of cognitive and behavioral techniques, no special focus on experiential techniques	Special focus on therapy relationship: Limited reparenting and empathic confrontation also contain validation strategies with a special focus on validation of traumatic childhood experiences as well as validation of emotions and needs, but not as explicitly as in the DBT protocol Empathic confrontation contains validation (esp. of needs and relationship to childhood experiences) of current dysfunctional mode-driven behavior and confrontation with problematic consequences and the need for change Skills are not trained directly Special focus on experiential techniques (esp. imagery rescripting and chair-dialogs) and therapy relation techniques Mode-specific use of cognitive and behavioral techniques
Analysis of problem behavior	Chain analysis according to the DBT model for each type of problem behavior; hierarchy of problem behaviors; focus on obvious and threatening problem behaviors such as suicide attempts, self-harm and impulsive behavior, focus on emotions and triggers as well as on consequences of behavior, no focus on needs	Analysis with cognitive or experiential techniques according to the mode model, mostly for problematic situations which lead to emotional suffering and frustration of needs; no specific hierarchy, focus both on obvious problem behaviors, but also on “hidden” problem behaviors such as avoidance or surrender, focus on emotional needs and modes
Structure of the individual therapy session	Fixed structure with a “crisp beginning” involving a diary card, processing of topics according to the DBT goal hierarchy, focus on emotions	No fixed structure specification, flexible hierarchy depending on the dominating mode and frustrated needs
Group therapy and structure of the group session	Group therapy is essential ingredient of DBT. Structure: Homework and goal-related opening and closing round, teaching of skills from the DBT modules with a fixed manual; preferred use of cognitive and behavioral therapeutic techniques	Group therapy is not mandatory, but has shown to be helpful in BPD patients. Structure: Begin with safety imagery, topics are covered depending on the dominating mode; designed as “group family” to create corrective experiences; preferred use of experiential and limited reparenting techniques
Dealing with self-injury	Fixed procedures according to protocol based strategies, top priority in goal hierarchy; self-injuries are usually discussed with behavioral analysis before other issues are addressed	No fixed structure specification, and need not be treated with first priority (only if highly threatening); therapeutic intervention is directed at the trigger mode
Dealing with emotional problems	Comprehensive psychoeducation in the modules for emotion regulation; mindfulness and acceptance of emotions; teaching and training of specific emotion regulation skills, decision on whether one should act according to or opposite to the emotion; emotion processing with the help of emotion protocols (more cognitive approach)	Promotion of safe experiencing of emotions; explaining aversive emotions and problems in emotion regulation within the mode model, especially in the beginning extrinsic emotion regulation through therapist according to the mode-specific goals, focus on needs (e.g. “What do I need when I'm sad?”); focus on experiential interventions, mainly imagery rescripting and chair dialogs, aims at developing corrective experiences
Development of the working alliance	Therapist as a “coach” of the patient; therapeutic team at eye level with patient, dialectical formation of working alliance with warmth, empathy, acceptance and validation on the one side and pushing for change on the other	Therapist acts to a limited extent as a good parent with “limited reparenting,” i.e., meeting needs of patient that were frustrated in childhood; use of the working alliance for changing modes and to experience emotions and relationships in a safe way
Mindfulness training	Central role; non-judgmental attitude is promoted	Not included in ST
Skills training in distress tolerance	High priority; psychoeducation, development of a skills chain for stress regulation to prevent problem behaviors, reality accepting skills to ease emotional pain	Limited use, mainly for emergency situations in the beginning of therapy

## Application

In this passage we will describe a case example of a woman with BPD and present the main strategies regulating emotions first from a DBT and then from a ST perspective.

### Case example

Mona, a 23-year old, overweight woman, comes to psychotherapy and reports: “I just cannot deal with my emotions, my moods shift so rapidly, no one is able to follow. I do not even understand myself. I guess, that is why I just cannot have a normal relationship. With my last boyfriend I had so many fights. I always thought he would leave me for another woman. I just could not trust him. I had so many rage attacks and threw things at him. And then he really left me. He said, he just could not stand it anymore….Well, and since then, I just do not want anyone close to me anymore, besides my little sister. It just does not work with me.” Asked directly for her symptoms, she reports cutting with razorblades about once a week (“That happens often when I am in an emotional chaos.…I do not care about the scars. I am ugly anyway”), daily binge eating in the evening and about three times a week smoking cannabis (“This just helps to calm down, when I feel lonely, sad or guilty”), suicidal ideas (“I often think, my life is a mess anyway and only pain. If I was dead, this all would stop and I would have peace and silence. I would not feel guilty and ashamed anymore. Nobody would miss me. I tried it four times with pills, but it did not work.”), social withdrawal and inactivity (“Most of the day I lie in bed. I really do nothing. I am a loser”). She also suffers from disturbing intrusive memories and nightmares, where she relieves physical and emotional abuse from her father and stepmother, but also from the death of her older sister. The sister died 19-year-old of a heroin overdose, when Mona was 15 years. Mona feels guilty that she did not help her. With regard to her biography she reports further: “My father drank a lot of alcohol. He was very impulsive, violent-tempered, often shouted at us and beat us. We all had panic, when he came home. My real mother was caring and warm-hearted, but she was also afraid of him and could not protect us. She died from cancer, when I was seven. My stepmother was also addicted to alcohol. She was very moody, sometimes she was nice, but then, and you could never tell when and why, she got angry, insulted and beat us. My elder sister was the only one, who was there for me. But when she began to take drugs, she became very unreliable and I was totally lost. For my younger sister I was the ‘mom,’ since nobody was there. She is still living with my parents. I cannot forgive myself that I left her there.”

### DBT perspective

#### DBT case concept

After a thorough assessment of Mona's presenting problems and her biography, the therapist educates Mona about BPD as a disorder of the emotion regulation system by using the biopsychosocial model: The precipitation factors were a history of invalidation, physical and emotional abuse by her parents in combination with a high emotional sensitivity. Mona has skills deficits in emotion regulation, in particular in dealing with grief and sadness (death of mother and sister, breaking up of partner), mistrust (expectations to be betrayed), anger (rage attacks), guilt (own behavior toward elder sister before her death, insufficient present support for her younger sister), and shame (own body, being mentally ill, abusing substances, disturbed eating behavior, inactivity). Both the externalizing behavior and internalizing problem behaviors (suicide attempts, self-injury, binge eating, drug use, social withdrawal, and inactivity) serve the avoidance or attenuation of aversive emotions and the associated physical symptoms of tension and pain. The patient also has skills deficits in the areas of self-management, mindfulness and metacognition, interpersonal behavior, and stress-tolerance. The therapist explains that DBT will focus on the acquisition of functional emotion regulation skills in the four modules, so that Mona can gain more control on her behaviors and in her life in general. The therapist uses a broad range of validation strategies to communicate acceptance and emphasize the understandability of Mona's behaviors and emotions. At the same time he motivates her to learn new strategies and pushes for change (dialectical balance of acceptance and change strategies).

The therapist sets up an intensive psychotherapy program with the following elements: individual therapy including telephone coaching, skills training group, case management by social worker, occupational therapy, and exercise therapy (Nordic walking).

#### Target hierarchy of problem behavior and goals for therapy

The therapist explains the DBT hierarchy of problem behaviors and Mona agrees with him on the following target hierarchy:

1. Suicidal behavior with intoxications
2. Severe self-injury with razor blades
3. Drug consumption
4. Binge eating
5. Social withdrawal,
6. Physical inactivity,
7. Economics.

They agree on the following goals and agreements for therapy:

- Regular attendance at therapy including all elements of the treatment program
- Practice of emotion regulation skills, especially learning new ways to tolerate and deal with grief, guilt, and shame
- Practice of distress-tolerance skills to prevent dysfunctional behavior
- Practice mindfulness and interpersonal effectiveness skills
- Preparation of a non-suicide decision
- Self-injuries must be medically cared of and be examined by a behavioral analysis
- Daily use of the DBT Diary Card to track problem behaviors as well as skill use, discussion at the beginning of each individual session
- Abstinence of drugs
- Practice of structured eating
- Developing a daily movement program
- Developing a perspective for education and work rehabilitation.

#### Understanding problem behavior and learning new skills

**Figure 1:**
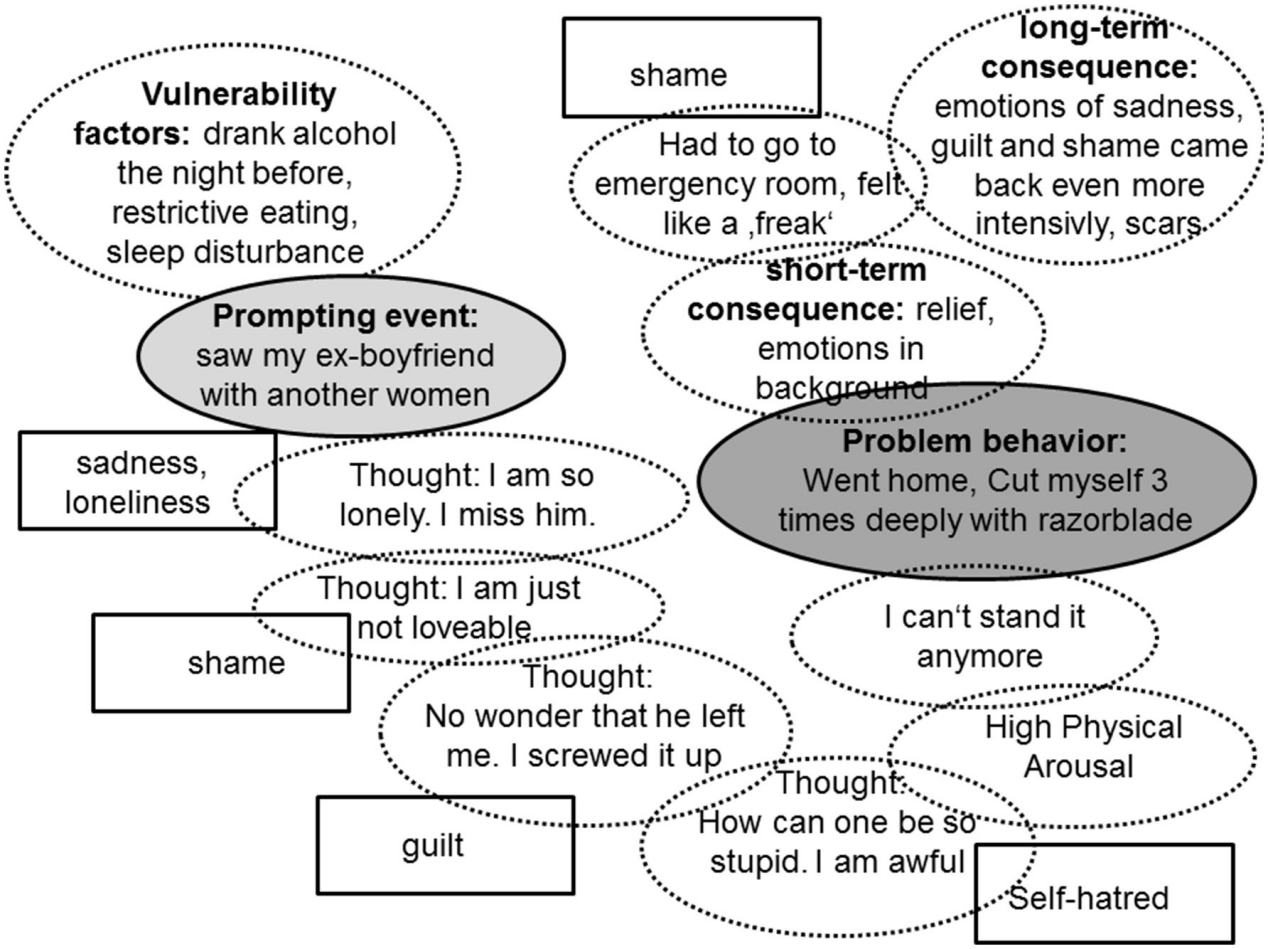
**Chain analysis of a self-injury**.

With behavioral analyses and chain analyses Mona learns to understand her own behavior, what it is caused by, why it is maintained and what consequences follow. Figure 1 shows a chain analysis of a serious self-injury (problem-behavior) after Mona saw her ex-boyfriend with another women (prompting event). Mona and the therapist work out emotional vulnerability factors, the emotions, thoughts, body reactions, and behaviors that follow the prompting event and end in the problematic behavior. Further they look at short-term and long-term consequences of the problem behavior.

After conducting the chain analysis they look for new skillful behavior to replace the problem behavior: The therapist explains that, when Mona is under such high tension, that she can't think clearly, she first needs to use her distress tolerance skill chain. She found out, that the best skill in such situations is to tip her head into a bucket full of ice-cold water or to do exercise (e.g., 20 sit-ups). When she has calmed down she needs to have a look at her emotions, accepting and without judgment. The therapist's validation strategies are very important for Mona to stop devaluation of her thoughts, behaviors and emotions. In the skill training group she learns to identify, describe and observe her emotions mindfully and about primary and secondary emotions. She also learns that she has a choice to act with or opposite to an emotion. Thus, she and her therapist go in many situations through each emotion involved and work out, if it is a primary or secondary emotion, if Mona wants to act with or opposite to it and how this behavior would look like. In the situation from the example chain analysis Mona and the therapist work out that sadness is the primary emotion, while guilt, self-hatred and shame are secondary emotions and result from judgments of the situation. With help of the therapist Mona decides that it would be good to act opposite to guilt, self-hatred and shame. Her action-urge from sadness is to withdraw from social contacts and to go in her bed. She anticipates that in a future situation this would end with her using drugs or binge eating. Thus, she decides to act opposite to this urge as well and plans to contact her friend Sarah in a future situation and ask her, if she can come around. Her new behavior plan is to self-validate herself, stop to blame and hurt herself, and on the contrary call her friend Sarah. The new skills are practiced intensively over and over again in individual therapy, group therapy and as homework.

Although, she does not like the skill of “radical acceptance” in the beginning, Mona finds out that this skill is especially important for her in situations she cannot change, e.g., in dealing with the loss of her mother and sister.

### ST perspective

#### Case conceptualization and psychoeducation with the mode model

**Figure 2:**
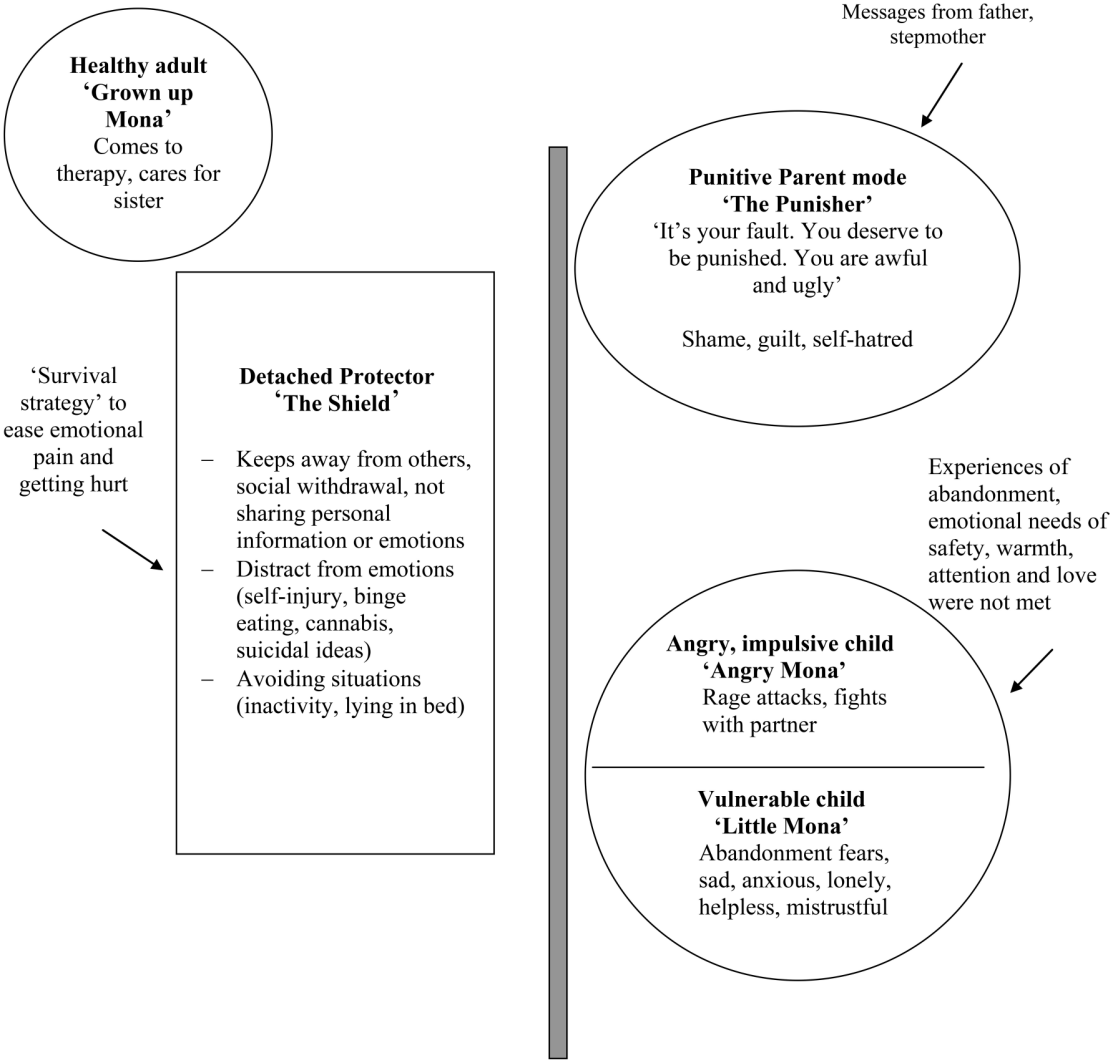
**Mona's mode model**.

After investigating Mona's current problems and her biography, the therapist develops an individual case conceptualization according to the mode model in interaction with Mona (see Figure 2). As usual in individual ST, Mona chose individual names for her modes: Mona's fears of being abandoned, feelings of mistrust, loneliness, sadness and anxiety are conceptualized in the vulnerable child mode (“little Mona”), her rage attacks and fights with the partner in the angry child mode (“angry Mona”). These modes developed since basic childhood needs have been frustrated and Mona has two times experienced a loss of her most important attachment figure. Self-devaluation, shame, guilt, and self-hatred refer to the punitive parent mode (“the punisher”), which developed probably due to experiences of aggression and insults from her father and stepmother. Early in life Mona developed “the shield,” her detached protector mode, as a survival strategy to protect herself from further emotional pain. In this mode she avoids getting close to others and distracts from intensive emotions or calms them down by self-injury, substance abuse, binge eating, social withdrawal, and sleeping. Her frequent mood-shifts and identity disturbances can be explained with rapid mode shifts. Mona's therapy attendance and her care for her sister are conceptualized in her healthy adult mode (“grown-up Mona”). The biographical context is brought into the case conceptualization with arrows (see Figure 1). The therapist helps Mona to foster her mode awareness and educates her about the general and mode-specific goals of ST. All of Mona's problems and symptoms are conceptualized and treated in terms of the modes involved.

#### Bypassing the detached protector mode

First the therapist works on Mona's detached protector mode, since it is very strong and rigid and blocks access to the child and parent modes. He starts by reviewing the pro and cons of this mode (see Table 3). Later the therapist proposes a chair dialog to better understand the “Shield.” He asks Mona to take place in a chair for the “Shield” and to answer to all his questions out of the “Shield's” view. He says: “Hello Shield. You're extremely important for Mona. I'd like to better understand you. Can you tell me, why you are here today?” He asks for the development of the mode (e.g., “Do you know, when you first came in Mona's life? Why did Mona need you?”). He validates Mona: “Oh yes, Mona told me, that she was often punished very harshly by her father, if she showed any feeling and that it was very painful, when her mother died. Nobody was there to help her. It was good, that you came to protect her.” After that the therapist aims to work out disadvantages of the “Shield” mode “I have the impression that something happened to little Mona last week, and that she feels overwhelmed and lonely. I really want to help her. But at the moment I can't see what it is, because you stand very strong in front of her and I cannot reach her. And I think this is not, what little Mona needs right now. What do you think?” Mona begins to cry and switches to the vulnerable child mode. The therapist takes another chair for “Little Mona” and places it next to his chair. She tells that she saw her ex-boyfriend with another woman and that she feels so sad, lonely and worthless. The therapist soothes and comforts her. A popping up of a punitive parent mode (“It's your fault. You screwed it up. You are just not lovable. And then you cut yourself. Loser”) is fought by the symbolic action of placing the chair of the “Punisher” out of the therapy room.

**Table 3:** **Pros and Cons of Mona's Detached Protector Mode**.

**Pros of “The Shield”**	**Con's of “The Shield”**
• Nobody can hurt me or abandon me • I have fewer conflicts with others • I do not have such awful feelings • I do not feel my needs and this is less painful, as I never get what I need anyway • I can control myself better e.g., do not have to cry • I feel safer • I have less awful memories • There is more silence	• I have no connection to others, I feel lonely and depressed • It feels empty and cold • It is boring • It still hurts and never stops • I do not have good contact with myself, I have no idea what I want in life. I have no control in my life • I have no idea about my emotions and needs, thus I can't fulfill my needs • I can't learn other strategies to deal with my problems and emotions

#### Healing the child modes and fighting the punitive parent mode

In the next phase of treatment dysfunctional child and parent modes are addressed with a strong emphasis on experiential techniques and therapy relationship techniques. An example is an imagery rescripting exercise on the physical abuse of the father. In the image Mona had accidently broken a bowl, the father shouts at her and loses his belt to beat her. The therapist enters the image, since he does not want Mona to relive the whole trauma. He steps between little Mona and her father to protect her and talks harshly to the father: “Stop at once. You are not allowed to beat little Mona. Nobody is allowed to beat children. It is quite normal that a bowl breaks from time to time. Mona has not done anything wrong!” Since the father gets even more aggressive the therapist has four police men enter the image and arrest the father. Mona sees how he is brought to jail. Asked for her feelings and needs, little Mona tells the therapist that it is good, that her father cannot harm her anymore, but that she still feels lonely and that she misses her mom, who died 3 months ago. And that she does not know where to go. The therapist listens to little Mona and soothes her. Finally, he takes her and her sisters to their aunt Mary, who Mona likes very much. At the end of the image aunt Mary reads Mona and her sisters “Pooh, the Bear.”

#### Strengthing the healthy adult mode

More imagery rescripting exercises of other adversive childhood memories are performed and with the course of therapy Mona herself in the healthy adult mode can comfort and soothe little Mona in the rescripting part. Also Mona and the therapist perform several chair dialogs in which Mona understands her contradicting emotional processes. She understands why she can feel guilty (punitive parent), angry (angry child), and sad (vulnerable child) at the same time. She learns to recognize and reduce her punitive parent mode including her feeling of guilt, self-hatred, and shame and to experience and validate the needs of her vulnerable child mode. First her therapist models these tasks for her, but with the course of therapy Mona can take over the role of her healthy adult mode herself each time a little better.

## Empirical evidence and future directions

### Empirical evidence for DBT

A systematic review and a Cochrane Review summarize the evidence for the efficacy of DBT in the treatment for patients with BPD, which has been shown in several randomized controlled trials (Kliem et al., 2010; Stoffers et al., 2012). The main effects are reduction of suicidality, self-injuring and impulsive behaviors, therapy dropouts and inpatient admissions. DBT has also shown effect in treating BPD with several comorbidities and other psychiatric conditions such as substance misuse (Linehan et al., 1999, 2002; Dimeff and Linehan, 2008), eating disorder (Safer et al., 2001; Telch et al., 2001; Kröger et al., 2010), post-traumatic stress disorder (Steil et al., 2011; Harned et al., 2012, 2014; Bohus et al., 2013), or depression (Lynch et al., 2007).

Research on mechanism of change has revealed that experiential avoidance impedes the reduction of depression in DBT-treatment of BPD and thus should be targeted (Berking et al., 2009). Experiential avoidance was decreased better in DBT compared to Community Treatment by Experts in a randomized controlled trial (Neacsiu et al., 2014a). Neacsiu et al. (2010) showed that increasing use of DBT skills is a mechanism of change for suicidal behavior, depression, and anger control in the treatment of BPD. This study supports the skills deficit model for BPD. Also DBT as a transdiagnostic treatment of emotion dysregulation was superior to activities-based support group in decreasing emotion dysregulation, increasing skill use and decreasing anxiety, but not depression in patients with mood and anxiety disorders. Skill use mediated the changes (Neacsiu et al., 2014b). Thus, behavioral skills are likely a potent mechanism of change for emotion dysregulation across disorders. However, evidence is preliminary and more research in other disorders than BPD is needed. Moreover, there are more than 60 DBT-skills and we do not know whether some skills are more important and useful than others in general, whether this varies over psychiatric disorders (e.g., patients with eating disorders needing other skills than patients with social phobia) or individual needs, whether some skills are more suitable for specific situations than others or how an individual determines to “use the right skill at the right time” and whether it executes that skill. Although, DBT has been evaluated intensively in efficacy and effectiveness studies, there is limited research on specific mechanisms of change in DBT. Clarifying the mechanisms of change could lead to a more focused and effective treatment and improvement on emotion dysregulation.

### Empirical evidence for ST

Empirical studies indicate high effectiveness of ST in the treatment of BPD regarding decreases in all nine BPD symptoms, improvements in quality of live and high treatment retention rate (Jacob and Arntz, 2013; Sempértegui et al., 2013). But also for other PDs results are encouraging: In a Dutch randomized controlled trial including patients with non-BPD PD with a majority of cluster-C-PDs (avoidant, dependent, and obsessive compulsive) ST was superior to two comparison conditions (Bamelis et al., 2013). Promising results are also reported for depression (Malogiannis et al., 2014; Renner et al., 2016).

Research on mechanism of change is in its infancy in ST: With regard to the “limited reparenting” approach, scores of the therapeutic alliance both of patients and therapists were higher in ST when compared to transference-focused therapy in the treatment of BPD (Spinhoven et al., 2007). Low ratings at early treatment predicted dropout, whereas positive ratings of patients predicted clinical improvement. Thus, the therapeutic alliance in ST may serve to facilitate change processes underlying clinical improvement in patients with BPD. Other hints on mechanism of change come from the non-BPD-trial (Bamelis et al., 2013): Therapists in this trial were trained in two waves, with the second wave of therapists being trained mainly by practicing in role plays and the first wave therapist by lecture and video-watching. The second wave of therapists had significantly less drop-out and stronger effects than the first wave of therapists. Therapists of the second wave reported to feel better equipped for the treatment and to have integrated all techniques. It is hypothesized that these therapist felt more secure in experiential techniques and thus experiential techniques were used to a greater extent and that this might have led to a better outcome. Several studies showed that imagery rescripting as a stand-alone technique is successful in a broad range of psychiatric disorders, including post-traumatic stress disorder (Arntz et al., 2007; Grunert et al., 2007; Raabe et al., 2015), social phobia (Wild et al., 2008; Brewin et al., 2009; Wild and Clark, 2011; Nilsson et al., 2012; Frets et al., 2014), or depression (Wheatley et al., 2007; Brewin et al., 2009; Review in Arntz, 2012). Therapeutic techniques using imagery instead of verbalization probably have greater impact on emotions (Holmes et al., 2009). It might be assumed, that imagery rescripting is an important technique to facilitate change in ST, however empirical evidence to support this hypothesis lacks. Other techniques used in ST, such as chair dialogs or historical role play, call for further investigation. How all these techniques provided by ST and ST in general impact emotion dysregulation remains up to date unclear and needs further study. Also, it would be very interesting to compare the effects on emotion dysregulation of ST to DBT and other methods.

### Future directions

From this comparison of DBT and ST with respect to emotion regulation several questions arise calling for further research. Stated in a simplified manner, DBT argues that emotion dysregulation skills deficits are the key to psychopathology, while ST assumes that early maladaptive schemas and modes underlie psychopathology and emotion dysregulation is a secondary consequence. If it is hypothesized that a treatment which addresses the key underlying factors of psychopathology has better treatment effects, the empirical question is to understand what underlies psychopathology. A question that is complicated to test, since assessment methods that specifically assess these underlying constructs with high validity need to be developed first.

Other important questions address the mechanisms of change for each method, but also differences between the two methods. Above for each method putative mechanism of change are discussed, e.g., skill use and targeting experiential avoidance for DBT or therapeutic alliance and use of experiential techniques in ST. However, the therapeutic alliance also plays an important role in DBT and ST is also targeting experiential avoidance, while skill use and use of experiential techniques are more specific to one of the methods. The question of specificity in these processes is very interesting, since basic processes that overlap in both methods and unique factors might be revealed and enable improvement of psychotherapy in general. Both treatments offer a variety of techniques and features. Currently it is impossible to say which ones are the most relevant for change. Component-analysis-studies are needed to reveal the most important features.

Treatment trials comparing DBT and ST are completely lacking, thus it remains an open question if one of the two methods is superior in efficacy and if the two methods have different efficacy for different groups of patients or different problems.

## Suggested readings and further resources

For further information on *DBT* we suggest the recent manual from Linehan (2015a,b), and the chapter from Neasciu et al. for the transdiagnostic DBT treatment model for emotion dysregulation (Neacsiu et al., 2015). A meta-analysis on treatment effects for DBT in the treatment of BPD can be found in Kliem et al. (2010).

For further information on *ST* we suggest the original book on ST from Young et al. (2003), a detailed manual on the work with the mode model from Arntz and Jacob (2012) and the manual for treating BPD from Arntz and van Genderen (2009). Recent reviews summarize current research findings on ST for BPD (Sempértegui et al., 2013) and PD in general (Jacob and Arntz, 2013).

## Author contributions

EF, US, and AA planed the concept and design of the paper. EF wrote the first draft of the paper. US, AA, DM, and OB provided critical revisions both from DBT and ST perspective. All the authors edited and revised the paper.

### Conflict of interest statement

EF, US, OB and AA give trainings and/or published books on Dialectical Behavior Therapy and/or Emotion Regulation in Schema Therapy. The other author declares that the research was conducted in the absence of any commercial or financial relationships that could be construed as a potential conflict of interest.
